# Supplementary material for: The Raw Milk Microbiota from Semi-Subsistence Farms Characteristics by NGS Analysis Method
Source: Molecules. 2021 Aug 19;26(16):5029. doi: 10.3390/molecules26165029 (PMC8402136; doi:10.3390/molecules26165029)
Supplement: Supplementary file 1 [file molecules-26-05029-s001.zip › molecules-1290322-supplementary.pdf]

Supplementary materials

Supplementary Table S1. The results of read-filtering in sequencing data analysis

Sample name	Number of reads	Filtered reads	Unique reads after filtering	Chimeric reads	Unique chimeric reads	Reads in OTUs
A1	168 683	58 758	19 742	10 229	834	99 696
A2	33 765	13 872	4 310	876	120	19 017
A3	58 939	29 338	8 175	1 531	297	28 070
A4	56 823	18 856	7 509	3 980	358	33 987
A5	23 100	9 758	2 929	638	86	12 704
A6	7 332	3 473	751	16	7	3 846
A7	163 449	46 077	19 110	12 619	540	104 753
A8	87 180	39 584	10 600	1 484	244	46 112
A9	251 192	90 074	31 327	12 301	1 119	148 817
A10	250 333	95 845	31 574	8 828	1 000	145 660
B1	509 904	95 840	76 047	24 053	2 539	390 011
B2	315 461	96 282	50 417	12 631	2 820	206 548
B3	110 845	37 956	18 475	4 093	919	68 796
B4	413 580	120 550	65 014	24 170	4 607	268 860
B5	393 219	91 449	62 354	27 685	4 817	274 085
B6	527 183	96 780	81 317	27 539	3 110	402 864
B7	632 956	112 960	96 648	29 911	3 899	490 085
B8	440 116	96 519	69 652	18 051	3 184	325 546
B9	356 486	78 306	54 694	15 424	2 167	262 756
B10	447 209	90 802	69 477	25 472	3 127	330 935
C1	259 197	50 883	24 388	34 796	1 146	137 518
C2	238 601	59 402	22 261	29 526	750	149 673
C3	209 169	45 337	19 561	21 336	639	142 496
C4	251 070	56 351	22 896	34 821	891	159 898
C5	253 785	50 589	23 161	45 686	1 103	157 510
C6	284 205	71 630	27 888	33 327	1 191	179 248
C7	203 973	66 208	21 342	11 428	612	126 337
C8	222 106	69 722	22 413	18 353	791	134 031
C9	273 670	86 309	29 469	24 196	1 550	163 165
C10	203 897	68 295	21 146	15 977	676	119 625

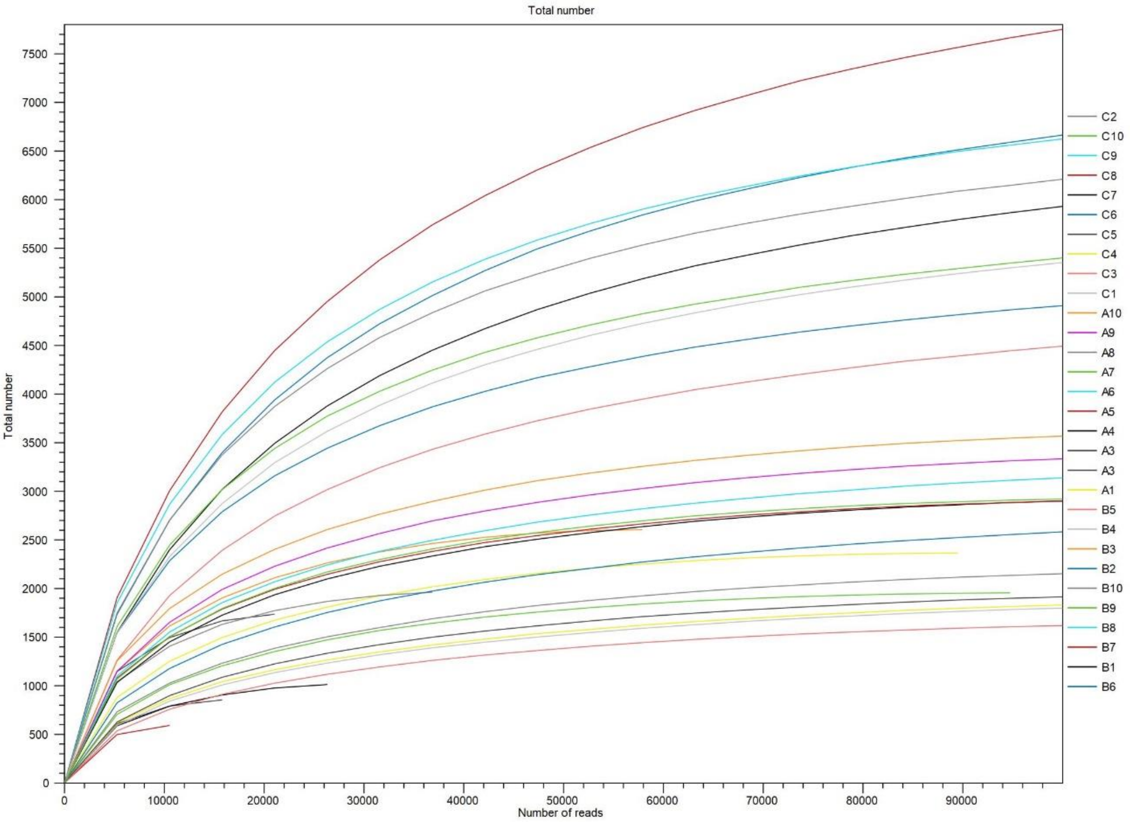

Supplementary Figure S1. Rarefaction curve used to calculate alpha-diversity
